# Telmisartan Prevents Alveolar Bone Loss by Decreasing the Expression of Osteoclasts Markers in Hypertensive Rats With Periodontal Disease

**DOI:** 10.3389/fphar.2020.579926

**Published:** 2020-11-11

**Authors:** Victor Gustavo Balera Brito, Mariana Sousa Patrocinio, Maria Carolina Linjardi, Ayná Emanuelli Alves Barreto, Sabrina CT Frasnelli, Vanessa Lara, Carlos Ferreira Santos, Sandra Helena Penha Oliveira

**Affiliations:** ^1^ Department of Basic Sciences, School of Dentistry, São Paulo State University (UNESP), Araçatuba, Brazil; ^2^ Multicenter Postgraduate Program in Physiological Sciences, Brazilian Society of Physiology, School of Dentistry, São Paulo State University (UNESP), Araçatuba, Brazil; ^3^ Department of Stomatology, Bauru School of Dentistry, University of São Paulo, Bauru, Brazil; ^4^ Department of Biological Science, Bauru School of Dentistry, University of São Paulo, Bauru, Brazil

**Keywords:** telmisartan, AT1 blocker, bone metabolism, spontaneous hypertensive rats, periodontal disease, osteoblast, osteoclast, cytokines

## Abstract

Periodontal disease (PD) is a prevalent inflammatory disease with the most severe consequence being the loss of the alveolar bone and teeth. We therefore aimed to evaluate the effects of telmisartan (TELM), an angiotensin II type 1 receptor (Agtr1) antagonist, on the PD-induced alveolar bone loss, in Wistar (W) and Spontaneous Hypertensive Rats (SHRs). PD was induced by ligating the lower first molars with silk, and 10 mg/kg TELM was concomitantly administered for 15 days. The hemimandibles were subjected to microtomography, ELISA was used for detecting tumor necrosis factor (TNF-α), interleukin-1β (IL-1β), interleukin-6 (IL-6), CXCL3, and CCL2, while qRT-PCR was used for analyzing expression of components of renin-angiotensin system (RAS) (Agt, Ace, Agt1r, Agt2r, Ace2, and Masr), and bone markers (Runx2, Osx, Catnb, Alp, Col1a1, Opn, Ocn, Bsp, Bmp2, Trap, Rank, Rankl, CtsK, Mmp-2, Mmp-9, and osteoclast-associated receptor (Oscar)). The SHR + PD group showed greater alveolar bone loss than the W + PD group, what was significantly inhibited by treatment with TELM, especially in the SHR group. Additionally, TELM reduced the production of TNF-α, IL-1β, and CXCL3 in the SHR group. The expression of Agt increased in the groups with PD, while *Agtr2* reduced, and TELM reduced the expression of Agtr1 and increased the expression of Agtr2, in W and SHRs. PD did not induce major changes in the expression of bone formation markers, except for the expression of Alp, which decreased in the PD groups. The bone resorption markers expression, Mmp9, Ctsk, and Vtn, was higher in the SHR + PD group, compared to the respective control and W + PD group. However, TELM attenuated these changes and increased the expression of Runx2 and Alp. Our study suggested that TELM has a protective effect on the progression of PD, especially in hypertensive animals, as evaluated by the resorption of the lower alveolar bone. This can be partly explained by the modulation in the expression of Angiotensin II receptors (AT1R and AT2R), reduced production of inflammatory mediators, the reduced expression of resorption markers, and the increased expression of the bone formation markers.

## Introduction

Periodontal disease (PD) is the most prevalent inflammatory disease worldwide, affecting the structures that support the teeth, including the gingiva, periodontal ligament, and alveolar bone (Pihlstrom et al., 2005). Without proper care, it can eventually lead to loss of teeth, resulting in a poor quality of life, impaired masticatory function, and speech impairment, and is therefore considered to be a public health issue. PD is initiated by gingival inflammation from the accumulation of a biofilm on the teeth. However, the progression of PD not only depends on microbial virulence, but also on the inflammatory response of the host, which plays a major role in tissue disruption. Comorbidities, such as hypertension, can significantly affect disease severity by increasing the inflammatory burden of the host, inducing alterations such as increased oxidative stress, causing endothelial dysfunction, and activating the renin-angiotensin system (RAS) (Paizan and Vilela-Martin, 2014).

The RAS is an endocrine system that governs electrolyte balance and blood pressure, and has a central role in the pathogenesis of hypertension. However, it is presently being studied due to its important role in inflammation. Briefly, angiotensinogen (Agt) is cleaved by renin to angiotensin (Ang) I, which is in turn cleaved to Ang II by the Ang-converting enzyme (Ace). The major effects of Ang II, including vasoconstriction, oxidative stress, increased blood pressure, and increased inflammation, are mediated via the type 1 receptor (At1r) (Paul et al., 2006; Capettini et al., 2012). Ang II type 2 receptor (At2r) has opposing effects, and induces vasodilation, reduces blood pressure, and attenuates inflammation (Paz Ocaranza et al., 2020). The non-canonical RAS axis comprises the Ang-converting enzyme 2 (Ace2), which directly cleaves Ang II to Ang 1–7, or Ang I to Ang 1–9, and the latter are further processed by Ace to Ang 1–7. Ang 1–9 can activate At2r, while Ang 1–7 binds to the Mas receptor (MasR). They have opposing effects on At1r signaling, which are mediated via counterregulatory mechanisms (Simões e Silva et al., 2013).

Substantial evidence indicates that the components of the RAS are locally expressed in numerous tissues, including the liver, kidneys, lungs, adipose tissues, adrenal tissues, and bone. These components act partially independently of systemic RAS, and are collectively known as local RAS (Shimizu et al., 2008; Giese and Speth, 2014). Previous studies have demonstrated that the components of the RAS are expressed in the local milieu of bones (Asaba et al., 2009; Izu et al., 2009; Yongtao et al., 2014; Zhang et al., 2014), and *in vitro* studies have demonstrated that Ang II receptors are expressed in cultured osteoblasts and osteoclasts (Asaba et al., 2009; Izu et al., 2009).

Telmisartan (TELM) is as potent At1r blocker that is clinically used to treat hypertension. It has numerous advantages over the other angiotensin receptors blockers (ARB), including a high volume of distribution, longer half-life, and consequently long-lasting effects, and can therefore be administered in single daily doses (Deppe et al., 2010; Frampton, 2011). TELM also acts as a partial agonist of peroxisome proliferator-activated receptor-γ (Fujimura et al., 2013; Kakuta et al., 2014). The effects of TELM on bone metabolism have already been studied; however, the results of these studies are controversial. Aydoğan et al. (2019) observed that TELM has no effects on the markers of bone turnover in patients with newly diagnosed stage I hypertension. However, another study by Ma et al. (2010), demonstrated that treatment with TELM reduces the bone loss induced by rosiglitazone in ovariectomized spontaneous hypertensive rats (SHRs). On the contrary, Birocale et al. (2016) observed that treatment with TELM negatively affected the bone quality of male SHRs.

We have previously reported that SHRs suffer from more severe periodontal inflammation (Bonato et al., 2012), and the inhibition of the RAS can attenuate the resorption of alveolar bone (Dionisio et al., 2019; Oliveira et al., 2019). In this study, we aimed to evaluate the local bone metabolism of normotensive and hypertensive animals with PD following treatment with TELM, as the effects of TELM on bone metabolism are yet to be clearly understood.

## Materials and Methods

### Animals, Ethical Aspects, and Experimental Groups

Experimental protocols were in accordance with Brazil’s National Council for Animal Experiments Control and were approved by the Ethics Committee on Animal Use from the School of Dentistry of Araçatuba (Unesp) (Process FOA-00686-2016). A total of 66 age matched (10-week-old) male rats from Wistar and SHR strains (*Rattus norvegicus albinus*) from the Central Animal Facility of School of Dentistry of Araçatuba (Unesp) were used. Animals were housed in a controlled light, temperature, and humidity environment (12 h light/dark cycle, 22°C ± 2°C, and 55% ± 5%), and offered a standard pellet diet and drinking water *ad libitum*. Animals from each strain (Wistar, W; and SHR, S) were randomly divided into the following experimental groups: Control (C), animals with periodontal disease (PD) and animals treated with telmisartan with PD (Telm + PD).

### Telmisartan Treatment

Animals were treated with TELM; 10 mg/kg (Micardis^®^; Boehringer Ingelheim, São Paulo, Brazil) dissolved in phosphate-buffered saline (vehicle), administered by oral gavage once daily for 15 days, beginning 1 day before PD induction. As the study main goal was to evaluate the local RAS role in the inflammation-induced bone alteration, drug treatment protocol was based on literature evidences of the anti-hypertensive effectiveness and anti-inflammatory properties in the periodontium of 10 mg/kg TELM in the rodent model (Wienen et al., 2001; Araújo et al., 2013).

### Periodontal Disease Induction

PD was induced by ligature inserted around the lower first molars, kept for 15 days. Briefly, animals were anesthetized (ketamine and xylazine hydrochloride association, 80 and 10 mg/kg) and placed in ventral decubitus on a dental table for rodents, with oral retractors supported on incisive teeth. A 4-0 silk thread (Shalon; Goiânia, Goiás, Brazil) was wrapped around the first inferior molars, carefully pushed into the gingival sulcus, and knotted medially.

### Non-Invasive Blood Pressure Measurement

To confirm the hypertensive phenotype and treatment effectiveness, systolic blood pressure (SBP) was verified by tail plethysmography (NIBP and PowerLab System; ADInstruments; Sydney, Australia) after the 15 days of PD and TELM treatment, and animals were considered hypertensive when SBP ≥150 mmHg (Potje et al., 2014).

### Euthanasia and Sample Harvest

On day 15 after PD induction, the animals were euthanized by inhalational anesthetic overdose (Isoflurane, Cristália, Itapina, SP, Brazil), the presence of bilateral ligature was evaluated, and animals in which it was absent were excluded from the study. To obtain samples for microtomography and histological analysis (*n* = 5/group), hemi-mandibles were surgically collected, and the right-sided specimens were stored in PBS at −20°C, and left-sided specimens were fixed in 10% buffered formaldehyde, respectively. To obtain samples for qRT-PCR (right-sided specimens) and ELISA (left-sided specimens) (*n* = 6/group) hemi-mandibles were surgically collected, immediately frozen in liquid nitrogen and stored at −80°C. Blood was also collected from animals, in heparinized flasks, and the plasma was separated by centrifugation (1,500 rcf; 10 min; 4°C) and stored at −80°C.

### Alkaline Phosphatase and Tartrate-Resistant Acid Phosphatase Activity Determination

Enzymes activities were determined by colorimetric assay in the plasma, as described by Fernandes et al. (2020). Briefly, for ALP assay reaction comprised 2.5 mM L *p*-nitrophenyl phosphate (pNPP), 2 mM MgCl_2_ and 25 mM glycine buffer (pH 9.4). TRAP assay reaction comprised 10 mM of pNPP, 50 mM sodium tartrate, 1 mM of *p*-hydroxy-mercury benzoate and 100 mM sodium acetate buffer (pH 5.8). Enzyme activity was calculated by the amount of hydrolyzed substrate (pNPP) per minute at 37 °C, and normalized by the total protein content, determined by the Lowry method (Lowry et al., 1951).

### Micro-Computed Tomography Analysis

Tomographic images from left hemi-mandibles were acquired using a SkyScan 1272 system (Bruker microCT; Kontich, Belgium) [70 kVp and 142 μA; 0.5 mm aluminum filter; 9 µm isotropic voxel; 1,100 ms exposure time, two frame averaging, and 180° rotation (0.5° rotation step)], and three-dimensionally reconstructed (NRecon software; v1.6; Bruker microCT). Alveolar bone loss was evaluated in the first molar region, and a region of interest (ROI) was standardized from defined anatomical points (upper limit: furcation roof; lower limit: proximal root apex; distal limit: second molar proximal root; proximal limit: first molar proximal root, and vestibular and lingual limits: limits of the alveolar bone). A volume of interest (VOI) was automatically delimited by the bone edges and the tooth volume exclusion. The bone percentage (%BV/TV), trabecular number (Tb.N), thickness (Tb.Th), and separation (Tb.Sp) were analyzed (Bouxsein et al., 2010).

### Gene Expression Analysis

Left hemi-mandibles were sectioned excluding the mandibular branch and the incisor tooth and cleaned from adjacent soft tissue. The specimens were then powdered in liquid nitrogen, and total RNA was extracted using TRIzol reagent (Invitrogen, Thermo Fisher Scientific; Carlsbad, CA, USA) following the manufacturer's instructions. RNA purity was assessed by 260/280 and 260/230 spectrophotometry ratio (satisfactory between 1.8–2.0, and 2.0–2.2, respectively). Samples were treated with DNAse I (Sigma-Aldrich), RNA was quantified (Quant-iT RiboGreen RNA Assay Kit, Invitrogen), and 2 µg of total RNA were reverse transcribed to complementary DNA (High Capacity RNA-to-cDNA^™^ Kit; Applied Biosystems, ThemoFisher Scientific; Foster City, CA, USA), according to manufacturer's instructions.

Gene expression analysis of bone markers was performed by quantitative real time-polymerase chain reaction (qRT-PCR), in StepOne PlusTM Real-Time PCR Systems, with TaqMan^™^ Gene Expression Assays (FAM fluorophore reporter/non-fluorescent quencher MGB) (Applied Biosystems, Thermo Fisher Scientific). Assay references are listed in Supplementary Table S1. Targets expression were normalized by *Actb* expression, as reference gene, and relative transcripts abundance was determined by the 2^(−∆∆Ct)^ method (Livak and Schmittgen, 2001).

### Immunohistochemical Assays and Analysis

Formaldehyde fixed mandibles were decalcified in 10% EDTA-buffered solution (Titriplex^®^ III; Merck Millipore; Burlington, MA, USA), for paraffin histological processing. Hemi-mandibles 3-µm thick sagittal sections were obtained (three to four tissue section per slide) and used for immunostaining of local RAS components. Briefly, tissue sections were deparaffinized, rehydrated, and were submitted to endogenous peroxidase blocking (Hydrogen Peroxide Block, DHP-125; Spring Bioscience Corp.; Pleasanton, CA, USA; 30 min incubation), and hot citric acid buffer antigen retrieval (10 mM citrate buffer, pH 6.0 at 55°C for 20 min, followed by cooling to room temperature for 20 min). Immunolabeling was performed by primary antibody incubation (described in Supplementary Table S2), and detected by Histofine^®^ Simple Stain^™^ kit (Nichirei Biosciences Inc.; Tokyo, Japan), followed by chromogenic substrate (3,3’-diaminobenzidine-tetrahydrochloride; Dako Corp., Carpinteria, CA, USA), according to manufactures instruction, and Harry’s hematoxylin was used as counterstaining. IHC assay batches (targets) were accompanied by negative control slides, which were submitted to the same procedures, but incubated with the antibody diluent solution only (Antibody Diluent, DHP-125; Spring Bioscience Corp.; Pleasanton, CA, USA), to ensure the absence of nonspecific staining in the tissue sections.

For IHC analysis, slides were blindly analyzed (*n* = 5/group) in 400× magnification bright-field microscopy (Olympus, BX53, Tokyo, Japan) in the region of interest (middle third of the first molar furcation region), and expression was based on the immunostaining pattern [negative (−); low staining (+); moderate staining (++); strong staining (+++)] (Oliveira et al., 2019). To compose the presented image boards, a representative section was photographed (Olympus, XC50, Tokyo, Japan), in the analyzed region of interest (400× magnification and detailed 1,000× magnification). Images were corrected for brightness and contrast, and black arrow indicate positive-stained bone cells.

### Cytokine Quantification

The left hemi-mandibles were sectioned from mandibular branch and incisor teeth and cleaned from adjacent soft tissues. The specimens were powdered in liquid nitrogen and subsequently homogenized with lysis buffer [cOmplete^™^ Protease Inhibitor Cocktail Tablet (Roche^©^), Tris-HCl 100 mM, NaCl 150 mM, Tween 20 1%, sodium deoxycholate 0.5%] in a tissue homogenizer (Omni TH, Omni International, Tulsa, OK, USA). The samples were centrifuged and in the recovered supernatant, and TNF-α, IL-6, IL-1β, CXCL3/CINC-2 and CCL20/MIP-3α were quantified by enzyme-linked immunosorbent assay (ELISA), with DuoSet^®^ ELISA (in order: DY510, DY506, DY501, DY540, and DY516; R&D Systems, Minneapolis, MN, USA). Results were normalized by the total protein content, determined by the Lowry method (Lowry et al., 1951).

### Statistical Analysis

Data are expressed as mean and standard error of the mean (SEM), and were analyzed by one-way ANOVA, followed by Šidak post hoc test, after being tested for normality distribution by Shapiro-Wilk test. Statistical differences are represented by brackets labeled by *p < 0.05, **p < 0.01, ***p < 0.001, and ****p < 0.0001, comparing Control vs. PD, PD vs. TELM + PD, and Wistar vs. SHR in the same experimental condition. All analysis was performed on Graph Pad Prism v7.0 (GraphPad Software Inc.; San Diego, CA, USA).

## Results

### Telmisartan Reverted the Spontaneous Hypertensive Rat Hypertensive Phenotype by Reducing the Systolic Blood Pressure

**FIGURE 1:**
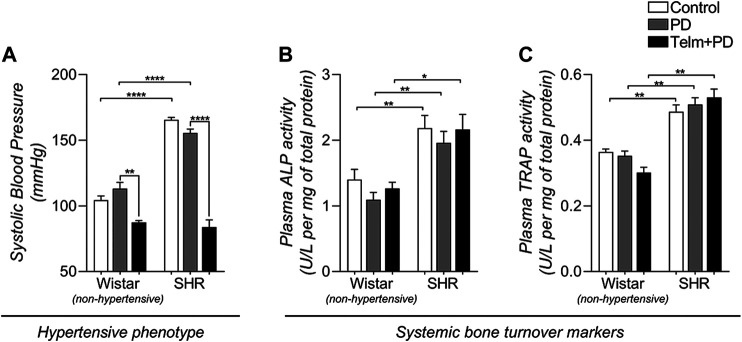
Systolic blood pressure (SBP) and systemic bone turnover biochemical markers of Wistar (non-hypertensive) and SHR with PD 15 days, treated with telmisartan. SBP **(A)** measured by tail plethysmography, and plasma activity of alkaline phosphatase, ALP **(B)** and tartrate-resistant acid phosphatase, TRAP **(C)** determined by enzymatic colorimetric methods. All graphs shown mean ± SEM (*n* = 6). Statistical difference are represented by brackets labeled by *p < 0.05, **p < 0.01, ***p < 0.001, and ****p < 0.0001, comparing Control vs. PD, PD vs. Telm + PD, and Wistar vs. SHR in the same experimental condition.

To confirm the TELM effectiveness of treated and non-treated animals, SBP was measured (Figure 1A). SHRs had increased blood pressure compared to Wistar rats, confirming the hypertensive phenotype, and PD induction did not significantly alter its values. As expected, TELM treatment led to a significant reduction in SBP in both the W and STelm + PD groups.

### Spontaneous Hypertensive Rat Presents Increased Alkaline Phosphatase and Tartrate-Resistant Acid Phosphatase Plasma Activity

ALP and TRAP activity were measured in the plasma as a bone forming and resorption biochemical marker (Figures 1B,C) to assess the proposed animal model's systemic bone dynamics and experimental conditions. SHR groups presented significantly higher ALP and TRAP plasma activity than the Wistar groups, suggesting increased bone turnover activity in the hypertensive strain. However, neither PD induction nor TELM treatment altered the systemic markers.

### Telmisartan Reduces Periodontal Disease-Induced Alveolar Bone Loss in Spontaneous Hypertensive Rats

**FIGURE 2:**
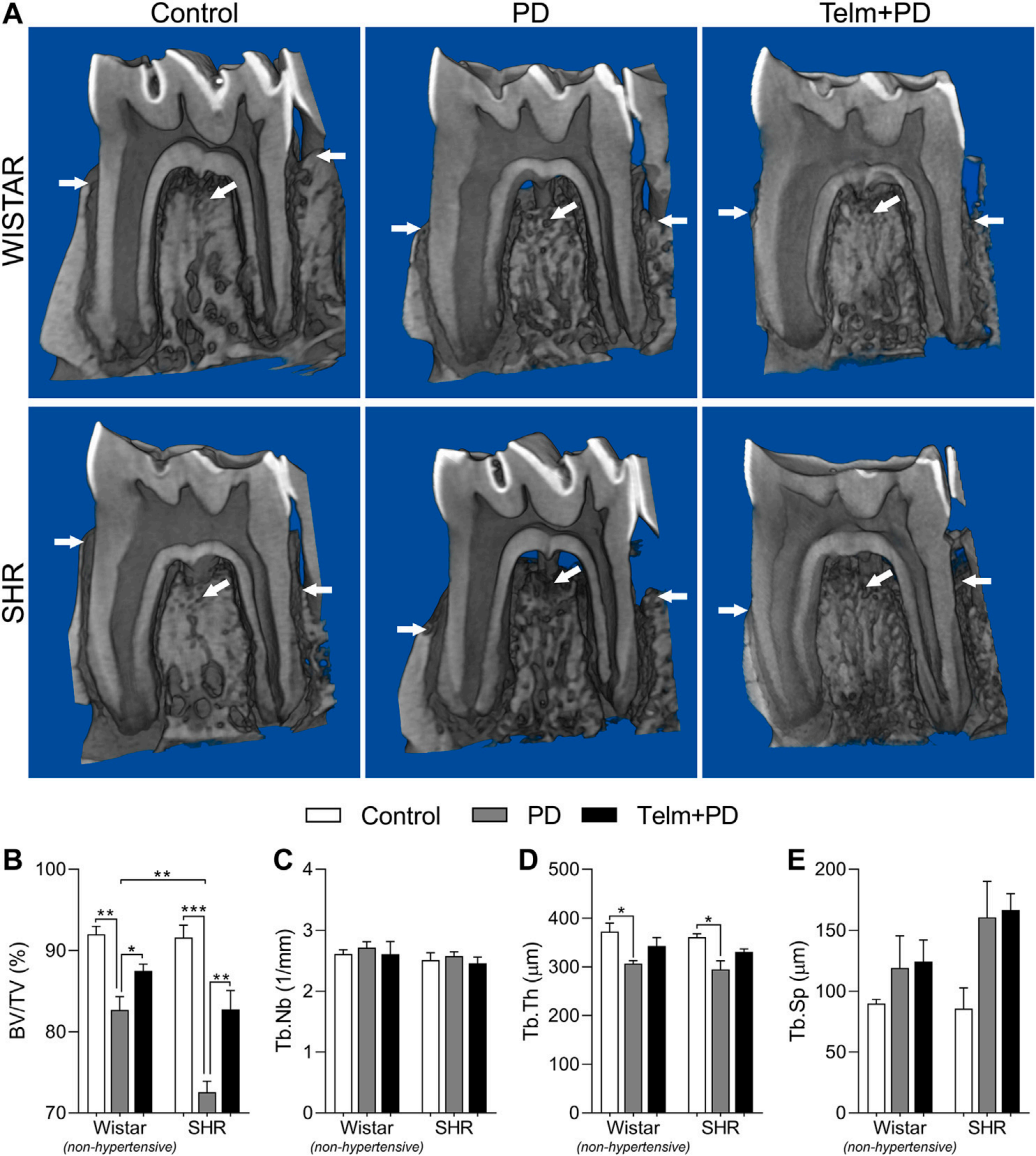
Microtomography analysis of mandible of Wistar (non-hypertensive) and SHR with PD 15 days, treated with telmisartan. Mandible three-dimensional reconstruction in lingual view with cut in the furcation region (white arrow indicate sites of significant bone resorption) **(A)**, and graphs showing %BV/TV **(B)**, Tb.Nb **(C)**, Tb.Th **(D)**, and Tb.Sp **(E)** as mean ± SEM (*n* = 5). Statistical difference are represented by brackets labeled by *p < 0.05, **p < 0.01, ***p < 0.001, and ****p < 0.0001, comparing Control vs. PD, PD vs. Telm + PD, and Wistar vs. SHR in the same experimental condition.

We first analyzed the effects of treatment with TELM on the loss of the alveolar bone in W and SHRs with PD, using microCT. The bone percentages and architectural parameters of the Wistar control (WC) and SHR control (SC) groups were similar, as expected (Figure 2A). However, the Wistar with PD (WPD) and SHR with PD (SPD) groups showed a significant bone loss, as evidenced by the reduced bone percentage (%BV/TV) (Figure 2B). It was noted that alveolar bone loss was more severe in the SPD group and was accompanied by the reduced trabecular thickness (Tb.Th) (Figure 2C), while the changes in the trabecular number (Tb.Nb) and trabecular separation (Tb.Sp) were not significant (Figures 2D,E). Treatment with TELM did not significantly alter the trabecular architecture but prevented bone loss in the Wistar with PD pretreated by TEMP (WTelm + PD) group, and the effect was more significant in the STelm + PD group.

### Telmisartan Reduces *Agt1r* Expression, and Increases the Expression of *Agt2r* in the Alveolar Bone

**FIGURE 3:**
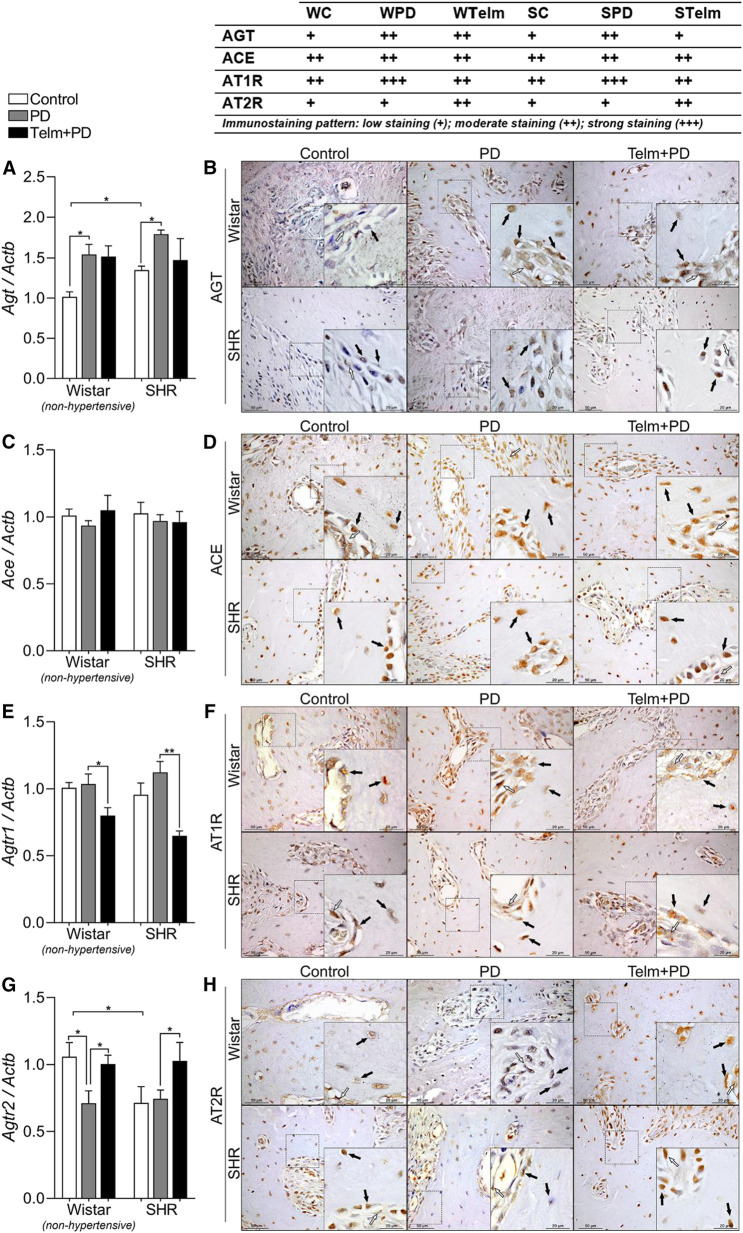
RAS components expression in mandibles of Wistar (non-hypertensive) and SHR with PD 15 days, treated with telmisartan. Respectively qRT-PCR and IHC for Agt **(A,B)**, Ace **(C,D)**, Agtr1 **(E,F)**, and Agtr2 **(G,H)**. Graphs show mean ± SEM (*n* = 6). Statistical difference are represented by brackets labeled by *p < 0.05, **p < 0.01, ***p < 0.001, and ****p < 0.0001, comparing Control vs. PD, PD vs. Telm + PD, and Wistar vs. SHR in the same experimental condition. Board shows representative images, and upper table presents the average immunostaining patter from each experimental group (*n* = 5). Black arrows point bone forming cells positive stained (osteoblasts and osteocytes), and white arrows indicate positive stained alveolar bone adjacent connective tissue.

To gain a better understanding of the participation of the local RAS in the protective effects of TELM, we analyzed the mandibular expression of the components of the RAS. The SC group had a higher *Agt* constitutive gene expression, and PD led to a significant increase in *Agt* expression, which was also confirmed by an increased immunostaining pattern in bone cells (black arrows) and adjacent connective tissue (white arrows) (Figures 3A,B).

Ace gene or protein expression was not significantly altered in the proposed experimental condition (Figures 3C,D). Agt1r expression was not significantly altered by PD, but TELM treatment significantly reduced its gene expression in WTelm and STelm, which was also observed in the lower immunostaining pattern in bone cells and adjacent connective tissue (Figures 3E,F). PD decreased *Agt2r* gene expression only in non-hypertensive animals (WPD, compared to WC), but TELM treatment increased its gene expression and immunostaining pattern significantly, observed in the bone cells and the adjacent connective tissue (Figures 3G,H).

**FIGURE 4:**
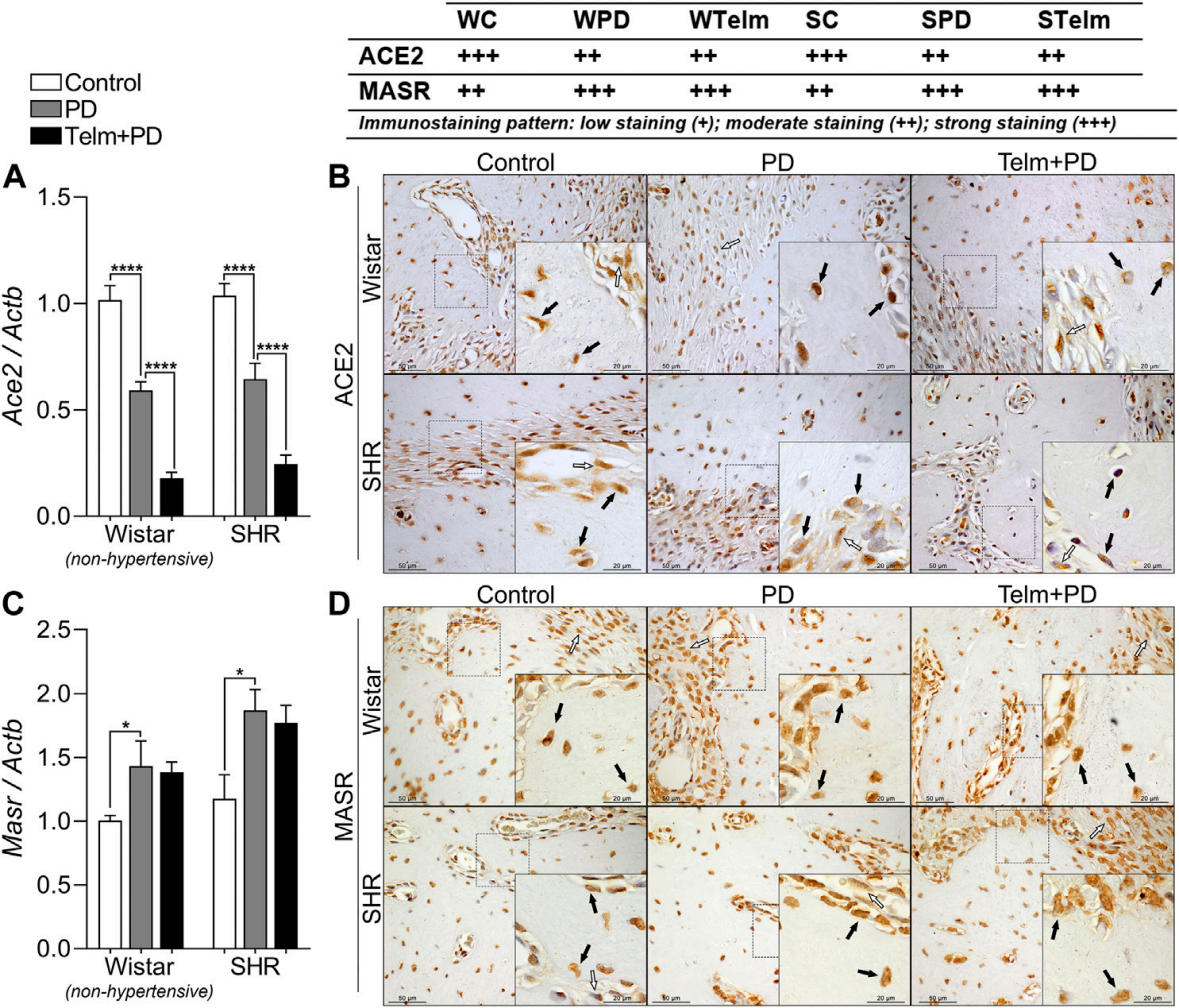
RAS components expression in mandibles of Wistar (non-hypertensive) and SHR with PD 15 days, treated with telmisartan. Respectively qRT-PCR and IHC for Ace2 **(A,B)**, and Masr **(C,D)**. Graphs show mean ± SEM (*n* = 6). Statistical difference are represented by brackets labeled by *p < 0.05, **p < 0.01, ***p < 0.001, and ****p < 0.0001, comparing Control vs. PD, PD vs. Telm + PD, and Wistar vs. SHR in the same experimental condition. Board shows representative images, and upper table presents the average immunostaining patter from each experimental group (*n* = 5). Black arrows point bone forming cells positive stained (osteoblasts and osteocytes), and white arrows indicate positive stained alveolar bone adjacent connective tissue.

Regarding the *Ace2/Masr* axis, W and SPD groups had decreased *Ace2* expression, compared to their respective controls, and TELM treatment led to further reductions in the target expression, observed by lower gene expression and lower immunostaining pattern (Figures 4A,B). In contrast, *Masr* expression was increased in W and SPD, compared to their respective controls, but TELM did not alter this response (Figures 4C,D).

### Telmisartan Reduces the Production of Inflammatory Cytokines in the Mandibles in Periodontal Disease

**FIGURE 5:**
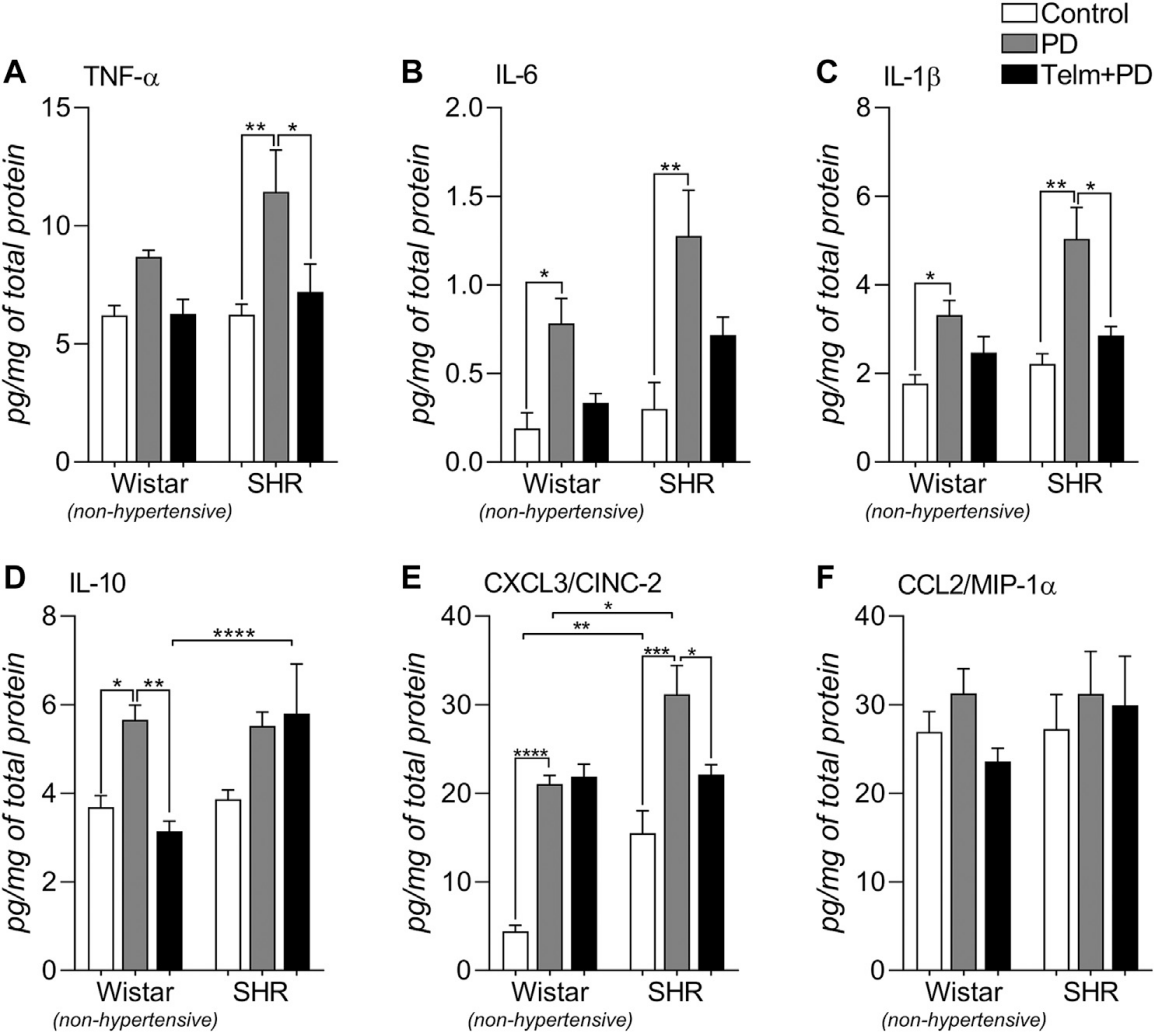
Inflammatory mediator production in mandibles of Wistar (non-hypertensive) and SHR with PD 15 d, treated with telmisartan. ELISA for TNF-α **(A)**, IL-6 **(B)**, IL-1β **(C)**, IL-10 **(D)**, CXCL3/CINC-2 **(E)** and CCL20/MIP-1α **(F)**. Graphs shown mean ± SEM (n = 6). Statistical difference are represented by brackets labeled by *p < 0.05, **p < 0.01, ***p < 0.001, and ****p < 0.0001, comparing Control vs. PD, PD vs. Telm + PD, and Wistar vs. SHR in the same experimental condition.

To assess the inflammatory response, we quantified inflammatory mediators’ production in the mandibles (Figure 5). PD led to a significant increase in the production of all analyzed pro-inflammatory cytokines, TNF-α, IL-6, and IL-1β, except for TNF-α in the WPD group, and TELM treatment was able to significantly reduce TNF-α and IL-1β production, only in the STelm + PD group than in the SPD group (Figures 5A,C). Regarding the anti-inflammatory cytokine IL-10, WPD presented an increased production, compared to its control, which was inhibited by TELM treatment, while in the SHR groups, it was not significantly altered (Figure 5D).

Regarding the analyzed chemokines, PD increased the neutrophil chemoattractant CXCL3, which was more significant in the SPD group than in the SC and WPD groups, and TELM only inhibited its production in the STelm + PD group (Figure 5E). CCL2 production, a macrophage chemoattractant, was not altered by PD or TELM treatment (Figure 5F).

### Telmisartan Increased the Mandibular Expression of *Runx2* and *Alp* in Wistar and Spontaneous Hypertensive Rats With Periodontal Disease

**FIGURE 6:**
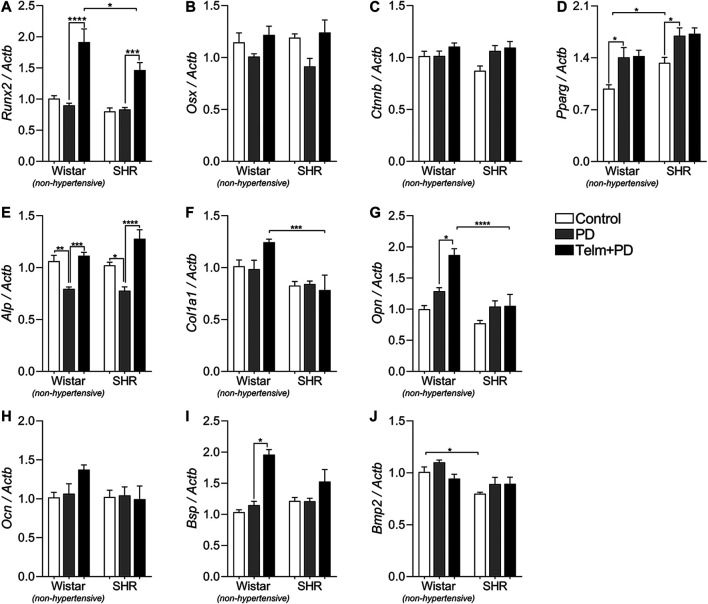
Bone formation markers expression in mandibles of Wistar (non-hypertensive) and SHR with PD 15 days, treated with telmisartan. qRT-PCR for *Runx2* **(A)**, *Osterix* **(B)**, *Catnb* **(C)**, *Pparg* **(D)**, *Alp* **(E)**, *Col1a1* **(F)**, *Opn* **(G)**, *Ocn* **(H)**, *Bsp* **(I)**, and *Bmp2* **(J)**. Graphs shown mean ± SEM (*n* = 6). Statistical difference are represented by brackets labeled by *p < 0.05, **p < 0.01, ***p < 0.001, and ****p < 0.0001, comparing Control vs. PD, PD vs. Telm + PD, and Wistar vs. SHR in the same experimental condition.

**FIGURE 7:**
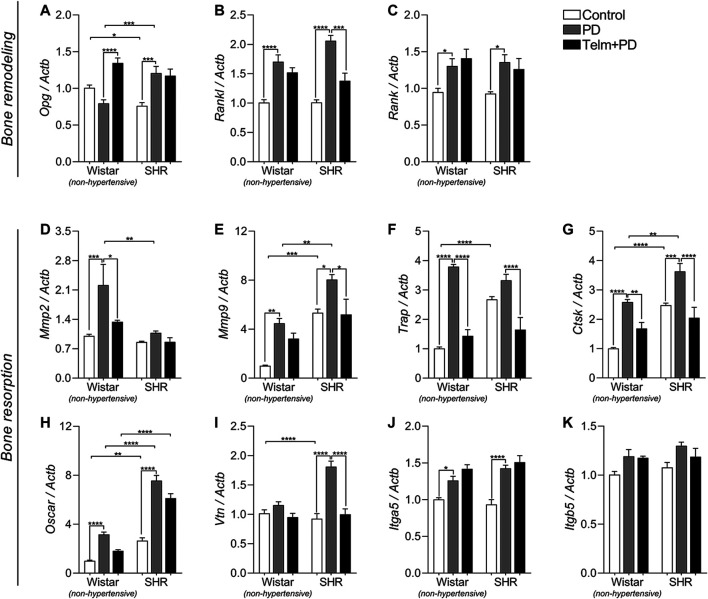
Bone remodeling and resorption markers expression in mandibles of Wistar (non-hypertensive) and SHR with PD 15 days, treated with telmisartan. qRT-PCR for *Opg* **(A)**, *Rankl* **(B)**, *Rank* **(C)**, *Mmp2* **(D)**, *Mmp9* **(E)**, *Trap* **(F)**, *Ctsk* **(G)**, *Oscar* **(H)**, *Vtn* **(I)**, *Itga5* **(J)**, and *Itgb5* **(K)**. Graphs shown mean ± SEM (*n* = 6). Statistical difference are represented by brackets labeled by *p < 0.05, **p < 0.01, ***p < 0.001, and ****p < 0.0001, comparing Control vs. PD, PD vs. Telm + PD, and Wistar vs. SHR in the same experimental condition.

To better understand of the local effect of TELM on the PD-induced bone response, we evaluated the gene expression of different bone markers (Figures 6, 7). First, on analyzed transcription factor expression, SC presented a higher constitutive Pparg expression than WC, and PD significantly increased its expression in W and SPD, while Runx2, Osx, and Ctnnb were not altered by PD induction. Interestingly, TELM treatment significantly increased Runx2 expression in W and STelm + PD compared to non-treated animals (Figures 6A–D).

**FIGURE 8:**
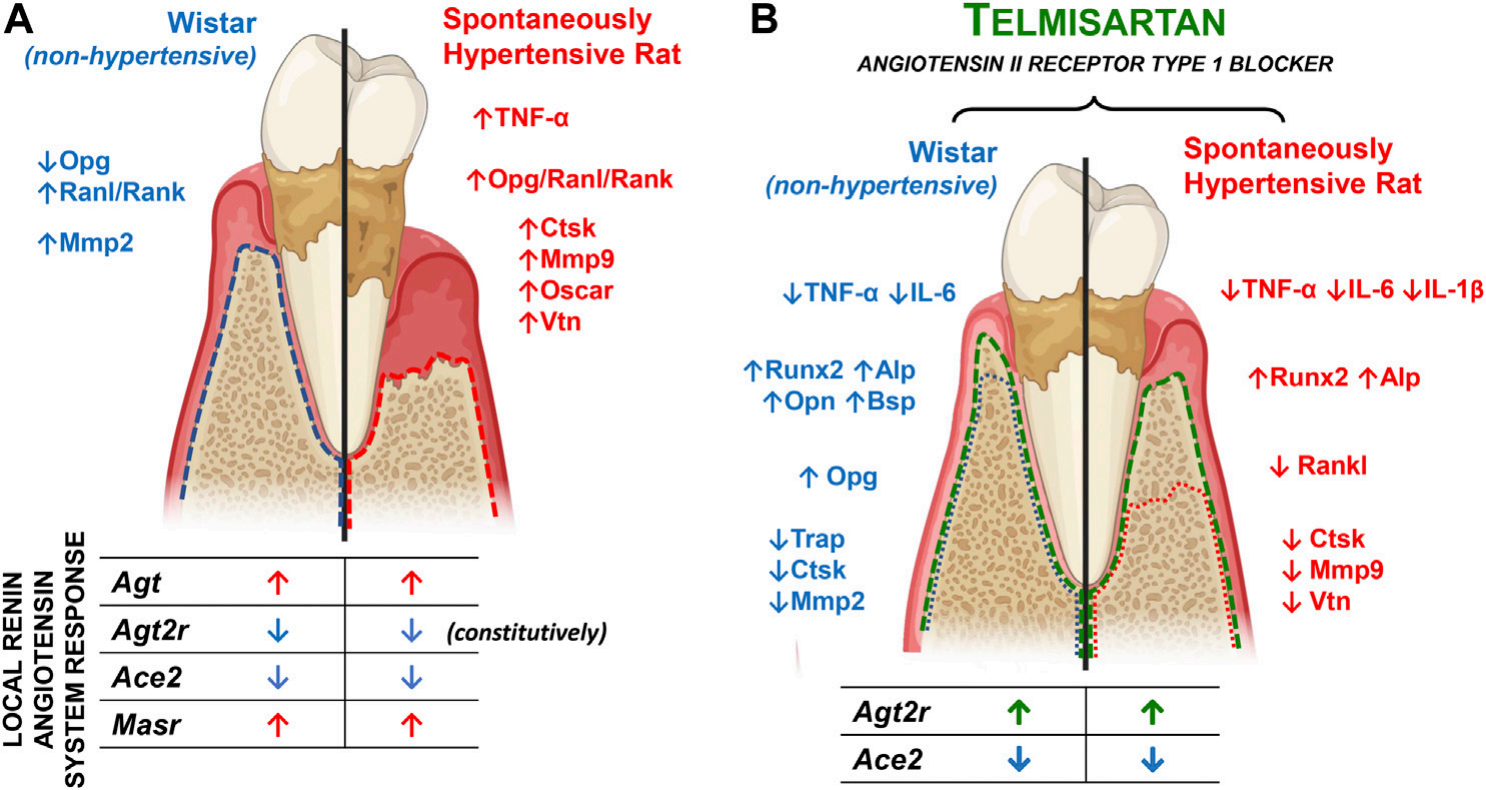
Main findings summary. **(A)** Main differences between Wistar (non-hypertensive) and SHR with PD. The blue and red dotted lines illustrate the alveolar bone loss in Wistar and SHR, respectively, the targets differentially expressed between the animal models are listed alongside, and the components of the local renin-angiotensin system altered by the PD are listed below. **(B)** Main effects of telmisartan in Wistar and SHR with PD. The green dotted line illustrates TELM protective effect in the PD-induced alveolar bone loss in Wistar and SHR, the related mechanisms are listed on the side, and the components of the local renin-angiotensin system altered by the treatment are listed below.

Regarding bone formation markers, only Alp expression was significantly reduced in the W and SPD groups compared to their respective controls, but TELM treatment was able to prevent this response (Figure 6E), and also increased the expression of Opn and Bsp in WTelm + PD, compared to WPD (Figures 6G,I). Additionally, *Col1a1* expression was higher in the WTelm + PD group than in the STelm + PD group (Figure 6E).

### The *Opg/Rankl/Rank* Axis Was Differentially Modulated by Telmisartan in the Mandibles of Wistar and Spontaneous Hypertensive Rats With Periodontal Disease

We then analyzed Opg/Rankl/Rank axis expression as essential markers of bone remodeling and dynamics (Figures 7A–C). At first, we noticed a decreased Opn constitutive expression SC, compared to the WC group, while Rankl and Rank expression were similar between the Wistar and SHR controls. PD significantly increased Rankl and Rank expression in the W and SPD groups, compared to their respective controls, while Opg was only increased in SPD, and not significantly altered in the WPD group (Figures 7A–C). TELM treatment, however, increased the expression of Opg only in the WTelm + PD group, and inhibited Rankl expression only in the STelm + PD group (Figures 7A,B), while Rank expression was not altered (Figure 7C).

### Telmisartan Reduced the Mandibular Expression of the Markers of Bone Resorption in Wistar and Spontaneous Hypertensive Rats With Periodontal Disease

Finally, bone resorption markers were evaluated, and we noticed a constitutive higher expression of *Mmp9, Trap, Ctsk, osteoclast-associated receptor (Oscar),* and *Vtn* in the SC group than in the WC group (Figures 7E–G,**I**). PD led to the increased expression of *Mmp9*, *Trap*, *Ctsk*, *Oscar*, and *Itga5* in the W and SPD groups, and it was more significant in the SPD group, than that of the WPD group, except for the Trap expression (Figures 7E–J). *Mmp2* expression was only increased in the WPD group, while *Vtn* expression only increased in the SPD group, compared to their respective control groups (Figures 7D,I). TELM treatment prevented the expression of these markers, except for *Mmp9, Oscar, and Itga5*, and only in the WTelm + PD group (Figures 7E,H,J).

## Discussion

The present study results demonstrated that the AT1R blocker, TELM, had a protective effect on PD-induced inflammation and alveolar bone loss in hypertensive animals by decreasing the production of cytokines and reducing the expression of osteoclast markers in hypertensive animals. The action of the local RAS in periodontal tissues has already been described and is associated with periodontal inflammatory damage (Santos et al., 2009; Santos et al., 2015; Dionisio et al., 2019; Oliveira et al., 2019). The SHR strain is known to present alterations in the systemic RAS (Schiffrin et al., 1984; Ohta et al., 1996; Gouldsborough et al., 2003) as well as intrinsic bone impairment associated with the hypertensive genotype and phenotype (Manrique et al., 2012; Landim de Barros et al., 2016; Tiyasatkulkovit et al., 2019). However, possible alveolar bone local RAS alterations and their association with increased susceptibility to inflammation-induced bone damage have not been shown.

After confirming the SHR hypertensive phenotype by increasing SBP (>150 mmHg) (Potje et al., 2014), we observed that TELM treatment was able to significantly reduce the SBP of Wistar and SHR, verifying the effectiveness of the chosen drug treatment regimen (10 mg/kg/day) to block the Ang II receptor type 1. According to the drug manufacturer information, this dose would represent approximately 1.25 times the maximum recommended human dose (80 mg/day) on an mg/m^2^ basis (Boehringer-Ingelheim, 2009). Other studies have also reported that this dose has significant antihypertensive and anti-inflammatory properties in rodents, corroborating our data (Wienen et al., 2001; Araújo et al., 2013). Additionally, to determine possible systemic bone alterations caused by PD or TELM treatment, which would affect the observed local response, plasma ALP and TRAP activity, were evaluated as biochemical markers of systemic bone turnover, bone formation, and resorption activity (Bauer et al., 2004; Halleen et al., 2006; Naylor and Eastell, 2012). However, these markers were not altered in the proposed experimental conditions. Interestingly, SHRs presented increased systemic ALP and TRAP activity, suggesting increased bone turnover. Altered systemic bone dynamics have already been reported in this animal strain (Tiyasatkulkovit et al., 2019), which, together with evidence of lower quality bone organic matrix production by SHR osteoblast precursors (Landim de Barros et al., 2016; Chaves Neto et al., 2018), would help to explain, in parts, the SHR increased susceptibility to bone loss.

We then aimed to evaluate the mandibular expression of the RAS components in hypertensive animals with PD in order to better understand this system’s role in the local bone response. We initially observed that *Agt* was constitutively expressed in SHRs, which increased after PD. *Agt* is the only precursor of all ANG peptides. Therefore, it is possible to suggest that the levels of active peptides of the RAS increase in inflamed bone tissues, primarily in hypertensive animals. *Agt* is initially converted to Ang I by renin (Lu et al., 2016); however, the expression of renin was not detected in our study, suggesting that the conversion of local Ang I is mediated by circulating renin, as previously observed (Oliveira et al., 2019). ACE, the enzyme that converts Ang I to Ang II, the most active RAS peptide, was not altered by PD. Numerous studies have previously suggested the role of this enzyme in bone physiology, demonstrating that the bone mineral density of patients increased after ACE inhibitor treatment, as the risk of fracture was reduced (Perez-Castrillon et al., 2003; García-Testal et al., 2006; de Vries et al., 2007; Kwok et al., 2012). Although constitutively expressed in both Wistar and SHRs, we could not associate the increased alveolar bone loss in hypertensive animals, nor the observed protective effects of TELM, to Ace expression modulations.

Similarly, the *At1r* receptor was constitutively expressed in both groups of animals, and PD did not alter Ang II receptor expression. However, as expected, TELM significantly reduced the expression of *At1r*, as it is an *At1r* antagonist, probably explained by *At1r* signaling inhibition. However, there was no difference in the normotensive and hypertensive animals’ responses, suggesting that its signaling was not related to the bone fragility differences between the Wistar and SHRs. Yu et al. (2019) demonstrated the *in vitro* anti-inflammatory effect of candesartan in human embryonic kidney epithelial cells (HEK lineage), possibly associated with reduced oxidative stress, independent of the *At1r* receptor. The present study’s results suggest that other mechanisms could have been involved in the protective effect of TELM on the bone. The protective effect of TELM observed herein could have been associated with the amount of cytokine production in the local lesion. The expression of *At2r*, which has already been associated with anti-inflammatory effects and tissue repair in different animal models (Namsolleck et al., 2014; Terenzi et al., 2017), was reduced following the induction of PD only in the W group and was similar to that of hypertensive animals following the induction of PD. However, the reduction in the expression of *Atr* was reversed by TELM in Wistar rats. TELM increased the expression of At2r in hypertensive animals, suggesting that the increased local production of Agt-derived peptides is associated with the inhibition of the *At1r* receptor. It could promote the activation of *At2r*, which increases the anti-inflammatory process and tissue repair in hypertensive animals. Previous studies have demonstrated that the activation of *At2r* antagonizes the harmful effects of AT1R activation (Kumar et al., 2002; Yang et al., 2012), and promotes the anti-inflammatory, anti-fibrotic, and anti-oxidative responses (Okada et al., 2006; Lu et al., 2015; Wang et al., 2017).

Although the expression of Ace remained unaltered during the inflammatory process, the expression of *Ace2* decreased after PD induction. The Ace2 enzyme is responsible for converting Ang II to Ang 1–7, which binds to *MasR*. The Ace2/Ang (1–7)/MasR pathway antagonizes the pro-inflammatory, pro-proliferative, and fibrotic effects induced by the Ace/Ang II/AT1R pathway, and is a novel target for the treatment of hypertension (Jiang et al., 2014). Yang et al. (2013) demonstrated that the expression of ACE increases in SHRs and that the expression of Ace2 is reduced in cardiac tissue compared to that in normotensive animals. It can thus be concluded that the expression of Ace2 depends on the specific tissue. The mechanism of action of Ace2 in the mandibular bone can be different from that of cardiac tissue. The latter may be different due to the differences in the cytokines released in the microenvironment. The induction of PD significantly reduced the expression of *Ace2ACE*, perhaps due to the increased production of cytokines; however, the expression of *MasR* increased in W and SHRs, which agreed with the results of other studies that demonstrated a lower *Ace2*/*Ace* ratio under inflammatory conditions (Hanafy et al., 2011; Sodhi et al., 2019). The increased expression of *MasR* suggested that a compensatory mechanism could have opposed the inflammatory process as an attempt to protect or induce tissue repair in the alveolar bone. However, further studies are necessary to understand the underlying mechanism better. Several studies have demonstrated that TELM increases the expression of the Ace2/MasR axis (Soler et al., 2009; Sukumaran et al., 2012; Yi et al., 2012) in myocarditis, renal vasculature, and hepatic fibrosis, but not in the alveolar bone. Thus, the effect can be tissue- and inflammation-dependent. The results of *in vivo* and *in vitro* studies have demonstrated that the ACE2/Ang 1–7/MasR axis positively modulates the activity of osteoblasts and inhibits the activity of osteoclasts (Abuohashish et al., 2017; Queiroz-Junior et al., 2019).

The inflammatory process induced by PD was mediated by releasing cytokines and chemokines in the mandibular bone. The expression of IL-6, IL-1β, and CXCL3 was increased by PD in normotensive and hypertensive animals. However, it is important to note that the increase in these mediators’ concentration was more significant in hypertensive animals. TNF-α is also involved in the inflammatory process in the mandibular bone of hypertensive animals, but not in normotensive animals, which confirmed an inflammatory potential in SHRs (Bonato et al., 2012). The cytokines TNF-α, IL-1β, and IL-6, are incredibly significant as they can stimulate osteoblasts by enhancing the expression of Rankl (Souza and Lerner, 2013). This mechanism may have been involved in inducing fragility in the mandibular bone of hypertensive animals after the induction of PD, as a large quantity of these cytokines was released, which increased the expression of Rankl and induced the activation of osteoclasts. The AT1R blocker, TELM, inhibited these cytokines’ production and the increased expression of Rankl strengthened this mechanism via the RAS receptor.

To gain a better understanding of the effect of TELM on alveolar bone resorption, we evaluated the gene expression profile of the markers of bone formation in the mandible. PD did not significantly change the expression of the transcription factors Runx2, Osx, and Ctnnb, but TELM significantly increased the expression of Runx2 in the W and SHRs with PD. Using ROS17/2.8 rat osteosarcoma cells and cell culture experiments, Nakai et al. (2015) demonstrated that Ang II inhibited the differentiation and mineralization of bones *in vitro* and reduced the expression of Runx2 via AGTR1. It is then suggested that the positive effect of TELM on the expression of Runx2 in animals with PD can be partially explained by its antagonistic effects on Ang II via AGTR1 in osteoblasts and their precursors.

*Pparg* is an important transcription factor that regulates bone metabolism. SHR presented a constitutively higher expression, which was also reported on *in vitro* osteogenic differentiation of bone marrow mesenchymal cells from young SHRs, before hypertension development, compared to cells from Wistar rats. This finding suggests an intrinsic alteration associated with the hypertensive genotype (Chaves Neto et al., 2018), which helps explain the SHR increased bone fragility since a sustained increase in Pparg activity can lead to bone impairments (Wan, 2010). In addition to the direct effect of *Pparg* on bone metabolism, it acts as an anti-inflammatory pathway, which inhibits NFkB and cytokine production (Korbecki et al., 2019). Studies demonstrating the Pparg expression profile in inflamed periodontal tissue are still limited, but we suggest that the increased Pparg expression in the PD group could represent a compensatory protective mechanism. TELM did not alter *Pparg* expression, despite its known partial *Pparg* agonist activity (Goyal et al., 2011), which may be explained by its already PD-induced increased expression, but further studies will be necessary to investigate this response further.

Analysis of the markers of bone formation revealed that PD only reduced Alp’s expression in both W and SHRs. Alp is one of the main enzymes involved in bone formation and plays an important role in matrix mineralization (Orimo, 2010). In this study, we observed that the reduction in Alp’s expression probably reflected the bone damage induced by PD, as evaluated by microCT. This expression could have resulted from the increased production of inflammatory mediators from immune cells during inflammation in the periodontium (Graves et al., 2011). TELM increased the expression of Alp, Opn, and Bsp in the normotensive animals, but only increased Alp’s expression in hypertensive animals. It is then suggested that some of the results observed herein may be related to the increased expression of Runx2, as previously discussed. The expression of Runx2 can modulate *Alp*, *Opn*, and *Bsp* gene expression, as reported by Jonason et al. (2009). Analysis of the bone matrix proteins, Col1a1 and Opn, revealed that their expression remained unaltered following the induction of PD. It is possible that matrix degradation had already taken place in the period evaluated in this study (15 days of PD), and it was therefore not possible to visualize this phenomenon (Almeida et al., 2015). However, TELM increased the expression of Col1a1 in normotensive animals and increased the expression of Opn, suggesting that the AT1R blocker TELM stimulated matrix synthesis by favoring the recruitment and adhesion of various cells, such as osteoblasts.

The Opg/Rankl/Rank axis is one of the primary regulators of osteoblasts and osteoclast activity. We observed that PD reduced the expression of Opg and increased the expression of Rankl and Rank, and the effect was more pronounced in hypertensive animals. As expected, PD induced the processes of alveolar bone resorption in these animals. TELM altered the expression of the Opg/Rankl/Rank axis components in a differential manner. In W rats, TELM treatment significantly increased the expression of Opg, which has an osteoprotective effect but reduced the expression of Rankl in the SHRs, which stimulates osteoclasts. These results suggest that TELM has a protective effect on bone resorption and reduced bone loss in hypertensive animals, possibly by inhibiting AT1R.

Analysis of the markers of bone resorption revealed that the expression of Trap, an enzyme responsible for the degradation of the mineralized matrix and an important marker of the activity of osteoclasts (Boyle et al., 2003), and Oscar, an IgG-type receptor with potent co-stimulatory functions in the differentiation of osteoclasts (Nemeth et al., 2011), significantly increased in the normotensive and hypertensive animals with PD, compared to that of their respective control groups. These results suggest that the inflammatory mediators released in the microenvironment can induce the differentiation and activation of osteoclasts, as Trap is a marker of osteoclast activity. It is important to emphasize that the differentiation of osteoclasts was more pronounced in hypertensive animals. Furthermore, the increase in the expression of Rankl in hypertensive animals induced the differentiation of osteoclasts in the W and SHRs. TELM significantly reduced the expression of Trap in both W and SHRs. However, the expression of Oscar remained unaltered in both the W and SHRs. TELM is thus more effective after maturation, but not in the earlier period during differentiation. Oscar has since been characterized as a costimulatory regulator of osteoclast differentiation (Kim et al., 2002).

Previous studies have demonstrated that the inhibition of matrix proteases reduces the inflammatory process and the destruction of the alveolar bone (Chen et al., 2016). In this study, PD significantly increased Mmp2, Mmp9, and Ctsk in normotensive and hypertensive animals. However, the expression of Mmp2 remained unaltered in hypertensive animals. This finding suggested minor participation of Mmp2 in the loss of the alveolar bone in SHRs, possibly due to the difference in the constitutive expression of Mmp2 between hypertensive and normotensive animals, and may consequently explain the exacerbated periodontal destruction in this model. The inhibition of AT1R reduced the expression of these proteases in animals with PD, and this effect was more pronounced in the SHRs. However, the expression of Mmp2 remained unaltered following treatment with TELM. This finding suggested that the expression of Mmp9 and Ctk depends on the components of the RAS, perhaps by inhibiting AT1R. AT1R modulates the signal by indirectly inducing the connection between Ang II and AT2R, which is responsible for the inhibitory effect on the expression of Mmps. Previous *in vitro* and *in vivo* studies have demonstrated that Ang II can stimulate the expression of Mmps via AT1R in different animal models, such as models of osteoblastic differentiation (Nakai et al., 2015). Furthermore, other authors have demonstrated that this inhibition is tissue-dependent (Okada et al., 2009; Okada et al., 2010; Araújo et al., 2013).

Vitronectin (Vtn) is an integrin that mediates cellular adhesion to the extracellular matrix, cellular migration, and cell-cell interactions (Barczyk et al., 2010). The expression of Vtns significantly increased after the induction of PD in hypertensive animals than in normotensive animals. TELM reduced this response, and the results suggested that the increased expression of Vtns depends on AT1R. Lakkakorpi et al. (1991) demonstrated that Vtns play an essential role in regulating osteoclasts and have a possible role in the permanence and migration of these cells. Steffensen et al. (1992) characterized the distribution of Vtns in the periodontium (marginal gingiva, periodontal ligament, endosteum, and periosteum). Although the results are speculative, this suggests that Vtns participate in the PD-induced loss of the alveolar bone, and is one of the factors responsible for the large difference in response between the normotensive and hypertensive animals. The results of this study further suggest that Vtns are modulated via AT1R. The extracellular matrix protein, Vtn, is recognized by at least one of the four known receptors, namely, integrins αvβ1, αvβ3, αvβ5, and αIIbβ3 (Felding-Habermann and Cheresh, 1993). In the present study, PD increased the expression of Itga5, which was more pronounced in hypertensive animals than in normotensive animals. The expression of Itgb5 remained unaltered, which indicated that AT1R did not mediate the expression of Itga5. Moreover, treatment with TELM did not modulate the expression of these receptors in either of the species. Although some studies have demonstrated the role of different adhesion molecules in periodontal tissues (Ghannad et al., 2008; Barczyk et al., 2010), there is very little information regarding the exact role of these proteins in alveolar bone loss during PD despite the evidence of the involvement of integrins in bone physiology (Slany et al., 2014; El Azreq et al., 2015). Additionally, these results suggested the involvement of Itga5 in the loss of the alveolar bone in hypertensive animals with PD (Figure 8).

These results suggest that the AT1R blocker, TELM, had a protective effect on PD-induced loss of the alveolar bone in the proposed experimental model. This effect was possibly due to the reduction in osteoclasts’ markers and the increase in the production of cytokines, which favored the activation of AT2R in hypertensive animals.

## Data Availability

The raw data supporting the conclusions of this article will be made available by the authors, without undue reservation.
