# Corrigendum: Telmisartan Prevents Alveolar Bone Loss by Decreasing the Expression of Osteoclasts Markers in Hypertensive Rats With Periodontal Disease

**DOI:** 10.3389/fphar.2020.635927

**Published:** 2021-02-19

**Authors:** Victor Gustavo Balera Brito, Mariana Sousa Patrocinio, Maria Carolina Linjardi, Ayná Emanuelli Alves Barreto, Sabrina CT Frasnelli, Vanessa Lara, Carlos Ferreira Santos, Sandra Helena Penha Oliveira

**Affiliations:** ^1^ Department of Basic Sciences, School of Dentistry, São Paulo State University (UNESP), Araçatuba, Brazil; ^2^ Multicenter Postgraduate Program in Physiological Sciences, Brazilian Society of Physiology, School of Dentistry, São Paulo State University (UNESP), Araçatuba, Brazil; ^3^ Department of Stomatology, Bauru School of Dentistry, University of São Paulo, Bauru, Brazil; ^4^ Department of Biological Science, Bauru School of Dentistry, University of São Paulo, Bauru, Brazil

**Keywords:** telmisartan, AT1 blocker, bone metabolism, spontaneous hypertensive rats, periodontal disease, osteoblast, osteoclast, cytokines

In the original article, there was a mistake in Figure 3 as published. On Figure 3 panel F, an error was made when assembling the representative imagens on the board, and the same image was included twice for the SHR Control group and SHR Telm + PD group. The correct representative image for the SHR Control group was included and the corrected Figure 3 appears below.

**FIGURE 3:**
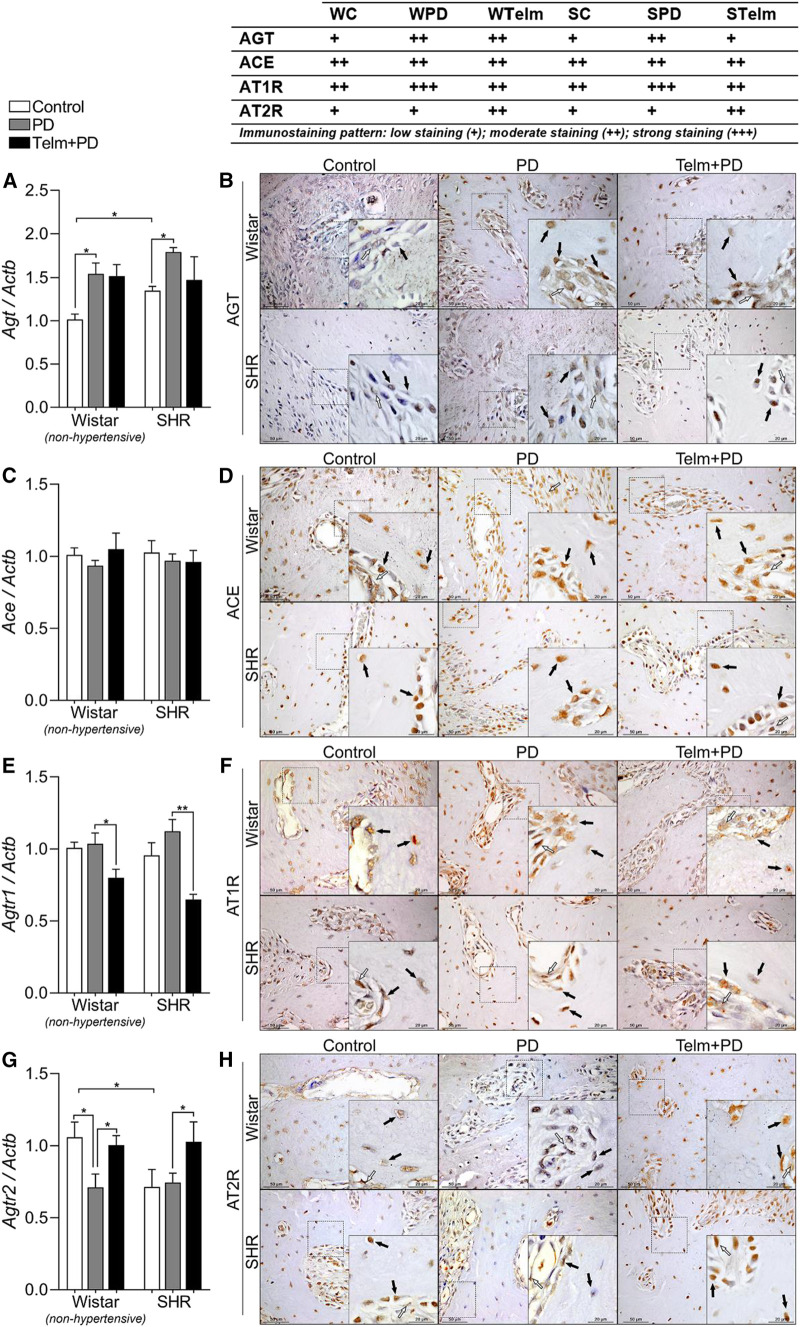
RAS components expression in mandibles of Wistar (non-hypertensive) and SHR with PD 15 days, treated with telmisartan. Respectively qRT-PCR and IHC for Agt **(A,B)**, Ace **(C,D)**, Agtr1 **(E,F)**, and Agtr2 **(G,H)**. Graphs show mean ± SEM (n = 6). Statistical difference are represented by brackets labeled by *p < 0.05, **p < 0.01, ***p < 0.001, and ****p < 0.0001, comparing Control vs. PD, PD vs. Telm + PD, and Wistar vs. SHR in the same experimental condition. Board shows representative images, and upper table presents the average immunostaining patter from each experimental group (n = 5). Black arrows point bone forming cells positive stained (osteoblasts and osteocytes), and white arrows indicate positive stained alveolar bone adjacent connective tissue.

The authors apologize for these errors and state that this does not change the scientific conclusions of the article in any way. The original article has been updated.

